# Not All Condemned to Die

**Published:** 1891-04-04

**Authors:** 


### Passing Topics.

### NOT ALL CONDEMNED TO DIE.

"On the —— inst., at Bournemouth, after a long illness, Nurse Charlotte, of the —— Hospital, aged 29, deeply regretted." Thus runs the short and touching announcement among the obituary notices in the newspapers, and thus ends a life which, we may be sure, was a useful one, and if we mistake not the writing between the lines, must have been sacrificed at the shrine of duty. It is an old, but it can never be an altogether trite reflection, that many a humble career is rife with all the elements of heroism. The path of duty is more often one of peril than is generally understood, and many a worker carries his life in his hand as he proceeds upon the round of his daily business.

Of course, there is plenty of mere unconcern, and we should not dream of confounding that with true heroism. The man who is too dull or too indifferent to estimate the danger he faces, necessarily lacks the best part of courage, and is no more to be commended for bravery than the wild animal which attacks the man, unconscious of the deadly power of the rifle he is carrying. That a large number of our nurses are fully entitled to the honours of heroism we have no doubt. Yet we question the accuracy of Sister Cecilia's statement "that every nurse, when she becomes a nurse, knows that she takes ten years off her life." Youth is prodigal, it is true, but we incline to the belief that this is a little too hard and mathematical a way of putting the truth Sister Cecilia intended to convey, and, without any loss of respect for the sentiment involved, we may fairly question whether any woman ever entered upon nursing with a knowledge of anything of the sort.

No doubt everybody who breathes the air of a hospital day by day is aware he incurs some risk of shortening life, but we must allow for possibilities. "Man thinks all men mortal but himself," is a fact we have all mastered more or less, and a widespread acceptance of this aphorism explains a great deal of daring. Happily not all heroes die young. Some survive to practice the virtues which come of experience and old age. It is always a pity to attempt to press a good point too far, and in this case, is it not enough to assert, as we may do unreservedly, that a majority of the women who undertake nursing are intelligent enough to comprehend the nature and the extent of the risks they run, and courageous enough to face them?

It was not ten years of her life, but forty, which, humanly speaking, poor Nurse Charlotte surrendered, and while some of her sisters will, like her, fall victims ere the fight is well begun, we may hope and believe that many will run the full course of life, none the worse bodily, and mentally all the better for their share in the good work of the world. We may bear in mind with advantage that the army of nurses is always in need of recruits, and that we must offer as inducements to the right kind of women, something more enticing than a certainty of bad health and early death. Sister Cecilia's dictum will probably be widely disseminated, and perhaps it will reach more ears than the corrections which have since been administered from other sources. Could we for a moment believe its truth to be capable of demonstration, we should say that our nursing methods and arrangements stand in need of instant revision.

Agricola.

